# *MTF1* genetic variants are associated with lung cancer risk in the Chinese Han population

**DOI:** 10.1186/s12885-024-12516-y

**Published:** 2024-06-28

**Authors:** Yujing Cheng, Chan Zhang, Qi Li, Xin Yang, Wanlu Chen, KunHua He, Mingwei Chen

**Affiliations:** 1 Department of Respiratory Medicine, The First Affiliated Hospital of School of Medicine of Xi’an Jiaotong University, Yanta District, No. 277, Yanta West Road, Xi’an, 710061 Shaanxi China; 2 Department of Blood Transfusion, The First People’s Hospital of Yunnan Province, The Afiliated Hospital of Kunming University of Science and Technology, Kunming, 650032 Yunnan China; 3 Department of Blood Transfusion, The First People’s Hospital of Qujing City, Qujing, 655099 Yunnan China

**Keywords:** Lung cancer, *MTF1* variants, Susceptibility, Stratification analysis, Demographic characteristics, Clinical features

## Abstract

**Background:**

Metal-regulatory transcription factor 1 (MTF1), a conserved metal-binding transcription factor in eukaryotes, regulates the proliferation of cancer cells by activating downstream target genes and then participates in the formation and progression of tumors, including lung cancer (LC). The expression level of *MTF1* is down-regulated in LC, and high expression of *MTF1* is associated with a good prognosis of LC. However, the association between *MTF1* polymorphism and LC risk has not been explored.

**Methods:**

The genotyping of *MTF1* Single nucleotide polymorphisms (SNPs) including rs473279, rs28411034, rs28411352, and rs3748682 was identified by the Agena MassARRAY system among 670 healthy controls and 670 patients with LC. The odds ratio (OR) and 95% confidence intervals (CI) were calculated by logistics regression to assess the association of these SNPs with LC risk.

**Results:**

*MTF1* rs28411034 (OR 1.22, 95% CI 1.03–1.45, *p* = 0.024) and rs3748682 (OR 1.24, 95% CI 1.04–1.47, *p* = 0.014) were associated with higher LC susceptibility overall. Moreover, the effect of rs28411034 and rs3748682 on LC susceptibility was observed in males, subjects with body mass index (BMI) ≥ 24 kg/m^2^, smokers, drinkers, and patients with lung squamous carcinoma (OR and 95% CI > 1,* p* < 0.05). Besides, rs28411352 (OR 0.73, 95% CI 0.55–0.97, *p* = 0.028,) showed protective effect for reduced LC risk in drinkers.

**Conclusions:**

We were first who reported that rs28411034 and rs3748682 tended to be relevant to increased LC susceptibility among the Chinese Han population. These results of this study could help to recognize the pathogenic mechanisms of the *MTF1* gene in LC progress.

**Supplementary Information:**

The online version contains supplementary material available at 10.1186/s12885-024-12516-y.

## Introduction

Globally, lung cancer (LC) is the second most frequently diagnosed cancer with an estimated 2.2 million (11.4%) new cancer cases and the leading cause of cancer death with an estimated 1.8 million (18.0%) deaths [1]. LC is the leading cause of cancer-related death in many developed countries [2]. In China, LC is also a type of primary cancer with high morbidity and mortality [3]. According to the LC statistics of China National Lung Cancer Research Center in 2015, the occurrence rate of LC in males is more than twice that of females (China's age-standardized mortality rate: 40.11 per 100,000 men and 16.54 per 100,000 women) [4]. Smokers, people exposed to smoke, oil fields, toxic occupational sites, people with family history, radiation exposure, and people with chronic lung diseases are all high-risk groups for LC screening [2]. In addition, genetic variants have been reported to explain about 12% to 21% of the heritability of LC [5, 6]. Previously, some association studies have identified multiple risk loci for LC development [7–9]. However, the genetic factors related to LC susceptibility have not been entirely recognized and there are still a large number of potential LC risk loci that need further study, especially in the Chinese population.

Metal-regulatory transcription factor 1 (MTF1) is a conserved metal-binding transcription factor in eukaryotes that binds to conserved DNA sequence motifs. *MTF1* regulates the proliferation of normal and cancer cells by activating downstream target genes and then participates in the formation and progression of tumors [10, 11]. *MTF1* has tumorigenic effects and promotes epithelial-mesenchymal transition (EMT) in ovarian tumor metastasis [12]. *MTF1* may be used as a diagnostic indicator of gastric cancer and is linked to a good prognosis [13]. *MTF1* is down-regulated in most cancers, including lung adenocarcinoma and lung squamous cell carcinoma, and higher expression of *MTF1* may predict a better prognosis for LC patients [14, 15]. Arsenic exposure is a worldwide health concern associated with an increased risk of lung cancer but arsenic trioxide (AsIII) is also an effective chemotherapeutic agent, Inactivation of ZnT1 or its transcriptional activator MTF1 resulted in considerable AsIII resistance [16]. A recent report showing that a novel Zn^2+^ chelator, LOR-253, can inhibit lung xenograft growth, proliferation and angiogenesis in association with changes in MTF-1 protein levels [17]. Gene expression profiles of A549 cultures treated with one of these water-soluble zinc ionophores, PCI-5002, reveal the activation of stress response pathways under the control of MTF-1 and heat shock transcription factors [18]. However, the association between *MTF1* polymorphism and LC risk has not been studied.

Here, we aim to study the contribution of *MTF1* gene variation to LC risk in the Chinese Han population, to investigate the interaction between *MTF1* gene polymorphism and demographic characteristics on LC risk, and to explore the relationship between *MTF1* polymorphisms and clinical features of LC patients.

## Methods

### Study population

A cohort of 1340 individuals from the First Affiliated Hospital of Xi’an Jiaotong University included 670 healthy controls and 670 LC patients with LC, all of whom were genetically unrelated individuals from the Han ethnic group. All patients were confirmed as primary LC by medical and histopathological examination according to the World Health Organization (WHO) classifications. Exclusion criteria included receiving radiotherapy or chemotherapy before the study, and a history of malignancy, chronic pulmonary diseases, and other serious organ diseases. All controls were randomly employed from the same hospital physical examination center. The control group met the inclusion criteria: 1) no personal or family history of malignant tumors, 2) no respiratory disease, infectious disease, or immune disease. These healthy controls were matched with LC patients on age, gender, body mass index (BMI), cigarette, and alcohol consumption status to minimize the effect of these confounders. Demographical and clinical data were collected through standard questionnaires and medical records. This study conformed to the Helsinki Declaration and was permitted by the Ethical Committee of the First People's Hospital of Yunnan Province (No. 2016LH036). All individuals signed informed consent forms.

### Genotyping

*MTF1* single nucleotide polymorphisms (SNPs) including rs473279, rs28411034, rs28411352, and rs3748682 were randomly selected for genotyping based on 1) the physical position and Variant table of the *MTF1* gene on the chromosome 1:37,809,574–37859592 through the e!GRCh38.p14 (http://asia.ensembl.org/Homo_sapiens/Info/Index) database, 2) minor allele frequency (MAF) > 0.01 from the dbSNP database for genotyping, 3) principles of MassARRAY primer design, 4) HWE > 0.05, and the detection rate for genotyping > 99.5% in our study, and 5) the genotype of these SNPs associated with the *MTF1* expression according to in silico analysis, as Supplementary Fig. 1. The GTEx Portal database (https://gtexportal.org/home/)[19], HaploReg v4.1 (https://pubs.broadinstitute.org/mammals/haploreg/haploreg.php)[20], and RegulomeDB (https://regulome.stanford.edu/regulome-search/)[21] bioinformatics tools were applied to predict the potential functions of these polymorphisms.

Peripheral blood samples (5 mL) of all participants were taken into EDTA vacutainer tubes, and genomic DNA was isolated by GoldMag DNA Extraction Kit (GoldMag Co. Ltd, Xi′an, China), and stored at –20˚C. The MassARRAY platform is based on MALDI-TOF (matrix-assisted laser desorption/ionization-time of flight) mass spectrometry in a high-throughput and cost-effective manner [22]. The genotyping steps for MassARRAY iPLEX were based on manufacturer’s protocol, as following: 1) DNA templates containing SNPs were amplified by PCR, and PCR products were treated with alkaline phosphatase to neutralize unincorporated nucleotides. 2) A single-base extension reaction was then performed to extend the PCR fragments by one base into the SNPs. 3) The resin purification reaction was performed, and the purified resin extension products were transferred to SpectroCHIP Assay using the purpose-built dispenser Agena Bioscience Nanodispenser RS1000. 4) Due to the different bases of polymorphism sites, the different terminal bases of extension products will lead to the difference of molecular weight after extension. The size of the product molecular weight was detected using MALDI-TOFmass spectrometry analysis. The Agena Biosicence Assay Design Suite V2.0 software (http://agenacx.com/online-tools) was used to design the extended primer. The MassARRAY Nanodispenser (Agena Bioscience, San Diego, CA, USA) and the MassARRAY iPLEX platform (Agena Bioscience, San Diego, CA, USA) were used to genotype, and then Agena Bioscience TYPER software (version 4.0) was used to analyze the data. The primers were detailed in Supplementary Table 1. Positive (Human Genome standard sample) and negative (H_2_O) controls were designed for each batch of genotyping experiments in the same 384-well plate. In addition, randomly selected 5% of samples were re-genotyping, with 100% consistency.

### Data analysis

The Kolmogorov–Smirnov test is a non-parametric test that assesses whether a sample comes from a population with a specific distribution, such as the normal distribution. Continuous variables was evaluated for normality using the Kolmogorov–Smirnov test. The Mann–Whitney U test, also known as the Wilcoxon rank-sum test, is a non-parametric test used to determine whether there is a significant difference between the distributions of two independent samples. It is an alternative to the independent samples t-test when the data does not meet the assumptions of normality. Continuous variables with non-normal distribution as median with interquartile range (IQR) were compared using Mann–Whitney U test. Categorical variables were compared between the study groups using χ^2^. The genotype frequency of these variants for Hardy–Weinberg equilibrium (HWE) was detected by the χ^2^ test. To evaluate the relationship of these SNPs to LC risk, logistics regression adjusted for variables such as age, sex, BMI, smoking, and drinking was used for determining the odds ratio (OR) and 95% confidence intervals (CI). False-positive report probability (FPRP) analysis was used to evaluate the noteworthy associations of the significant findings to reduce the numbers of false-positive findings [23]. FPRP threshold is 0.2, and the prior probability is 0.1, which is used to evaluate the significant association of significant findings. If the FPRP value is less than the preset threshold (0.2) at the prior probability is 0.1, it means that the false positive rate of the positive result is lower than the expected value, and the positive result is noteworthy. Furthermore, a multifactorial dimensionality reduction (MDR) analysis (https://sourceforge.net/projects/mdr) [24, 25] for the influence of SNP-SNP interaction on LC risk was performed by MDR 3.0.2. Using this method, multilocus genotypes are classified into high-risk and low-risk groups, effectively reducing the genotype predictors from n dimensions to one dimension. The new, one-dimensional multilocus genotype variable is evaluated for its ability to classify and predict disease status through cross-validation (CV). The pairwise linkage disequilibrium (LD) were measured by the Lewontin’s coefficient D’ using the Haploview v4.2 software and the correlation of *MTF1* haplotypes with LC risk was evaluated by logistic regression model. Data were studied by SPSS version 20.0 (SPSS Inc., Chicago, IL, USA), and *p* < 0.05 was significant. The statistical significance of the Bonferroni-corrected *p* values would be set at *p* < 0.05/(4 × 4 × 5).

## Results

### Study population characteristics

Table 1 displayed the frequency distributions of age, sex, smoking, alcohol consumption, and BMI between 670 patients with LC (61 years, male/female: 472/194) and their 670 matched non-cancer healthy controls (61 years, male/female: 464/206). The table also listed the clinical data of LC patients, including pathological type, stage, and lymph node metastasis. No significant differences in age (*p* = 0.371), sex (*p* = 0.547), smoking (*p* = 0.291) drinking (*p* = 0.229) status, and BMI (*p* = 0.993) between the two groups were observed. Adenocarcinoma accounted for 46.4% (311/670), followed by squamous cell carcinoma (31.6%, 212/670).

**Table 1:** Characteristics of patients with lung cancer and healthy controls

Variables	Cases	Control	*p*
N	670	670	
Age, years (median with interquartile range)	61 (53–68)	61 (53–68)	0.0.371^a^
≤ 50	121 (18.1%)	107 (16.0%)	
51–60	204 (30.4%)	207 (30.9%)	
61–70	250 (37.3%)	270 (40.3%)	
> 70	95 (14.2%)	86 (12.8%)	
Gender			0.547^b^
Male	476 (71.0%)	464 (69.3%)	
Female	194 (29.0%)	206 (30.7%)	
Smoking			0.291^b^
Yes	405 (60.4%)	386 (57.6%)	
No	265 (39.6%)	284 (42.4%)	
Drinking			0.229^b^
Yes	354 (52.8%)	332 (49.6%)	
No	316 (47.2%)	338 (50.4%)	
BMI, kg/m^2^ (median with interquartile range)	24.6 (22.2–27.1)	24.7 (22.5–26.6)	0.993^a^
≥ 24	387 (57.8%)	393 (58.7%)	
< 24	283 (42.2%)	277 (41.3%)	
Pathological type			
Squamous carcinoma	212 (31.6%)		
Adenocarcinoma	311 (46.4%)		
Missing	147 (21.9%)		
Stage			
I-II	291 (43.4%)		
III-IV	379 (56.6%)		
Lymph node metastasis			
Yes	313 (46.7%)		
No	343 (51.2%)		
Missing	14 (2.1%)		

*SD* standard deviation, *BMI* body mass index

*p*^a^ values were calculated from Mann–Whitney U test

*p*^b^ values were calculated from χ^2^ test

### Association between MTF1 SNPs and susceptibility to LC

The information on *MTF1* SNPs (rs473279, rs28411034, rs28411352, and rs3748682) was presented in Table 2, including Chr: position, role, allele, MAFs (> 0.05) of cases and controls, *p* for HWE (*p* > 0.05), and the potential function of these SNPs. Moreover, rs28411034 (OR 1.20, 95% CI 1.02–1.41, *p* = 0.032) and rs3748682 (OR 1.22, 95% CI 1.03–1.43, *p* = 0.020) tended to have increased LC susceptibility in the allele model. Here, none of the SNPs was significant after the Bonferroni correction.

**Table 2:** Basic information of candidate SNPs of *MTF1* in the study

SNP ID	Chr: Position (GRCh38)	Role	Allele	MAF		HWE *p*	OR(95% CI)	*p*	RegulomeDB	Haploreg4.1
				Case	Control					
rs473279	1:37,810,130	3'-UTR	T/C	0.284	0.278	0.082	1.03 (0.87–1.22)	0.703	eQTL/caQTL + TF binding / chromatin accessibility peak	SiPhy cons, Promoter histone marks, Enhancer histone marks, DNAse, Proteins bound, GRASP QTLhits, Selected eQTLhits
rs28411034	1:37,811,325	3'-UTR	A/G	0.324	0.286	0.108	1.20 (1.02–1.41)	**0.032**	eQTL/caQTL + TF binding / chromatin accessibility peak	SiPhy cons, Enhancer histone marks, DNAse, Proteins bound, Selected eQTLhits
rs28411352	1:37,812,907	3'-UTR	T/C	0.189	0.204	0.812	0.91 (0.75–1.10)	0.331	eQTL/caQTL + TF binding / chromatin accessibility peak	Enhancer histone marks, DNAse, Motifs changed, NHGRI/EBI GWAS hits, GRASP QTL hits, Selected eQTL hits
rs3748682	1:37,814,315	3'-UTR	C/T	0.325	0.284	0.296	1.22 (1.03–1.43)	**0.020**	eQTL/caQTL + TF binding / chromatin accessibility peak	Promoter histone marks, Enhancer histone marks, DNAse, Motifs changed, NHGRI/EBI GWAS hits, GRASP QTL hits, Selected eQTL hits

*p *values were calculated from *χ*^2^ test

Bold values are statistically significant (*p* < 0.05)

HaploReg v4.1 (https://pubs.broadinstitute.org/mammals/haploreg/haploreg.php), RegulomeDB (https://regulome.stanford.edu/regulome-search/)

*SNP* single nucleotide polymorphism, *Chr* chromosomal, *MAF* minor allele frequency, *HWE* Hardy–Weinberg equilibrium, *OR* odds ratio, *95% CI* 95% confidence interval, *eQTL* expression quantitative trait locus, *TF *transcription factor

By HaploReg annotation, we found that the possible function of these SNPs might be related to SiPhy cons, promoter/ enhancer histone marks, DNAse, motifs changed, proteins bound, GRASP quantitative trait locus (QTL) hits, and selected expression quantitative trait locus (eQTL) hits. The results of RegulomeDB displayed that these selected SNPs were related to eQTL/caQTL, transcription factor binding, and/or chromatin accessibility peak. Based on the GTEx Portal database, the genotypes of rs473279 (*p* = 4.55e-6), rs28411034 (*p* = 5.96e-6), rs28411352 (*p* = 6.88e-37), and rs3748682 (*p* = 7.05e-19) in *MTF1* were associated with the mRNA expression in lung tissues (Supplementary Fig. 1). These results might suggest that these polymorphisms may be involved in LC carcinogenic by affecting the expression or function of *MTF1*, which provides a theoretical basis for subsequent mechanistic studies.

The genotype frequency distribution of these SNPs between two groups was shown in Table 3. Genotypes distribution analysis showed that rs28411034 A-allele carriers (GA + AA genotypes) were more frequent in the case group than in controls (55.8% vs. 50.3%). Genetic model results indicated that rs28411034 was linked with increased LC susceptibility under the dominant (OR 1.26, 95% CI 1.01–1.56, *p* = 0.038) and log-additive (OR 1.22, 95% CI 1.03–1.45, *p* = 0.024) models. Moreover, rs3748682 C-allele carriers (TC + CC genotypes) were more frequent in cases than in controls (56.0% vs. 49.6%). *MTF1* rs3748682 was also related to the higher LC risk under the codominant (OR 1.28, 95% CI 1.02–1.60, *p* = 0.046), dominant (OR 1.30, 95% CI 1.05–1.62, *p* = 0.016), and log-additive (OR 1.24, 95% CI 1.04–1.47, *p* = 0.014) models. Here, none of the SNPs was significant after the Bonferroni correction.

**Table 3:** The association of *MTF1* genetic polymorphisms and lung cancer risk

SNP ID	Model	Genotype	Control	Case	OR (95% CI)	*P*-value	AIC	BIC
rs473279	Codominant	C/C	339 (50.8%)	341 (50.9%)	1	0.510	1867.3	1908.9
		C/T	287 (43.0%)	277 (41.3%)	0.96 (0.77–1.20)			
		T/T	42 (6.3%)	52 (7.8%)	1.24 (0.81–1.92)			
	Dominant	C/C	339 (50.8%)	341 (50.9%)	1	0.970	1866.6	1903
		C/T-T/T	329 (49.2%)	329 (49.1%)	1.00 (0.80–1.23)			
	Recessive	C/C–C/T	626 (93.7%)	618 (92.2%)	1	0.270	1865.4	1901.8
		T/T	42 (6.3%)	52 (7.8%)	1.27 (0.83–1.93)			
	Log-additive	–-	–-	–-	1.04 (0.87–1.23)	0.670	1866.5	1902.8
rs28411034	Codominant	G/G	333 (49.7%)	296 (44.2%)	1	0.078	1866.4	1908
		G/A	291 (43.4%)	314 (46.9%)	1.22 (0.98–1.53)			
		A/A	46 (6.9%)	60 (9.0%)	1.47 (0.97–2.23)			
	Dominant	G/G	333 (49.7%)	296 (44.2%)	1	**0.038**	1865.2	1901.6
		G/A-A/A	337 (50.3%)	374 (55.8%)	**1.26 (1.01–1.56)**			
	Recessive	G/G-G/A	624 (93.1%)	610 (91.0%)	1	0.160	1867.5	1903.9
		A/A	46 (6.9%)	60 (9.0%)	1.34 (0.89–2.00)			
	Log-additive	–-	–-	–-	**1.22 (1.03–1.45)**	**0.024**	1864.4	1900.8
rs28411352	Codominant	C/C	426 (63.6%)	439 (65.5%)	1	0.530	1870.2	1911.8
		C/T	215 (32.1%)	209 (31.2%)	0.95 (0.75–1.19)			
		T/T	29 (4.3%)	22 (3.3%)	0.73 (0.41–1.30)			
	Dominant	C/C	426 (63.6%)	439 (65.5%)	1	0.470	1869	1905.4
		C/T-T/T	244 (36.4%)	231 (34.5%)	0.92 (0.74–1.15)			
	Recessive	C/C–C/T	641 (95.7%)	648 (96.7%)	1	0.310	1868.4	1904.8
		T/T	29 (4.3%)	22 (3.3%)	0.75 (0.42–1.31)			
	Log-additive	–-	–-	–-	0.91 (0.75–1.10)	0.330	1868.6	1905
rs3748682	Codominant	T/T	336 (50.4%)	295 (44%)	1	**0.046**	1861.2	1902.8
		T/C	283 (42.4%)	314 (46.9%)	**1.28 (1.02–1.60)**			
		C/C	48 (7.2%)	61 (9.1%)	1.46 (0.97–2.20)			
	Dominant	T/T	336 (50.4%)	295 (44.0%)	1	**0.016**	1859.6	1896
		T/C–C/C	331 (49.6%)	375 (56.0%)	**1.30 (1.05–1.62)**			
	Recessive	T/T-T/C	619 (92.8%)	609 (90.9%)	1	0.200	1863.7	1900.1
		C/C	48 (7.2%)	61 (9.1%)	1.29 (0.87–1.92)			
		T/C	283 (42.4%)	314 (46.9%)	1.21 (0.97–1.50)			
	Log-additive	–-	–-	–-	**1.24 (1.04–1.47)**	**0.014**	1859.4	1895.7

*p* values were computed by logistic regression analysis with adjustments for age, gender, smoking, drinking and BMI

Bold values are statistically significant (*p* < 0.05)

*SNP* single nucleotide polymorphism, *OR* odds ratio, *95% CI *95% confidence interval, *AIC *Akaike information criterion, *BIC *Bayesian information criterion

### Association of MTF1 polymorphisms with the LC risk in subgroup analysis by demographic characteristics

**Fig. 1:**
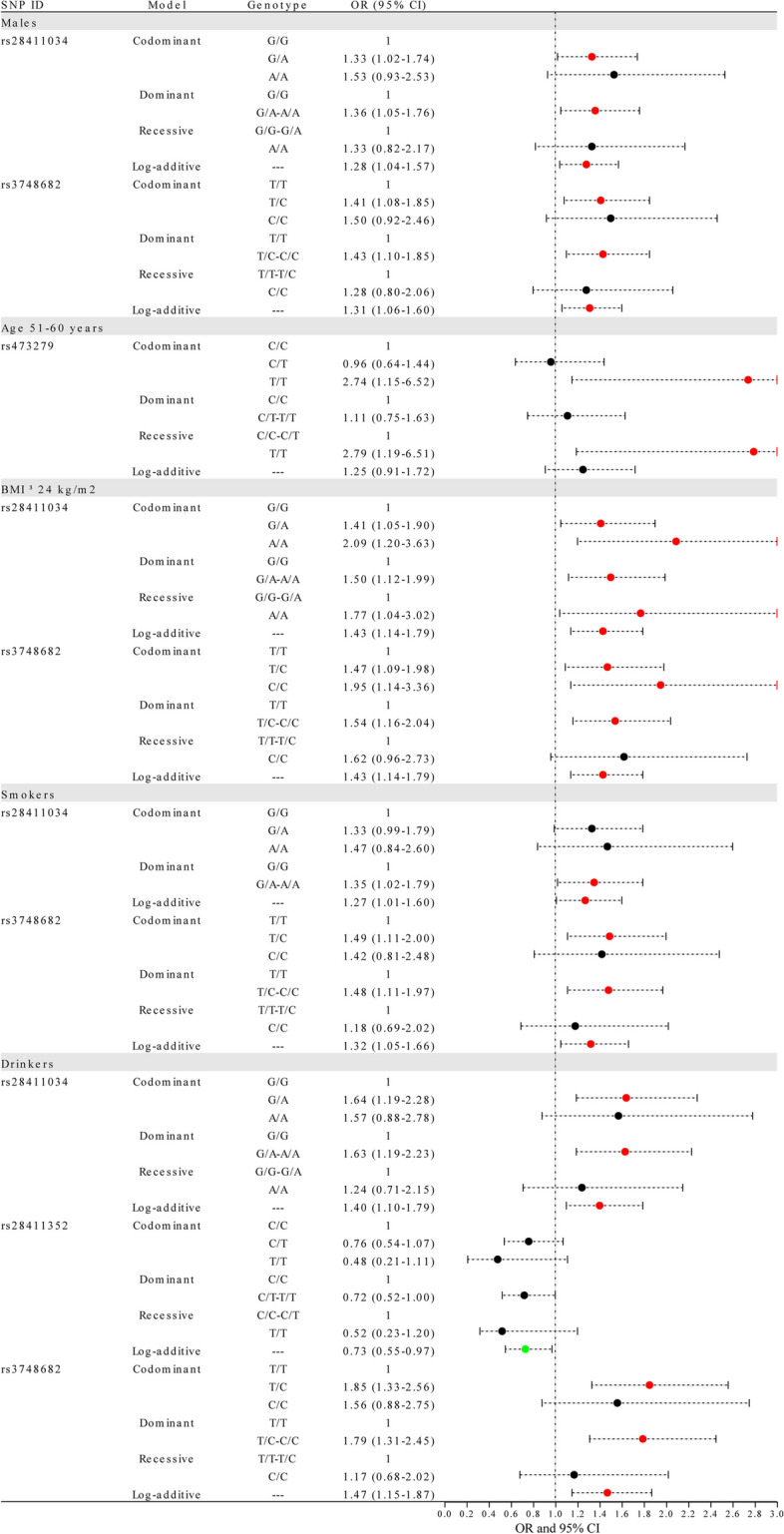
Forest map for the association of *MTF1* variants with the LC risk in subgroup analysis by demographic characteristics. The red dots denote the SNP/model associated with an increased risk of lung cancer, green dots represent the SNP/model related to a decreased risk, and black dots indicate the SNP/model with no significant correlation to lung cancer risk. Furthermore, the dots signify the OR values, while the bars represent the 95%CI. LC: lung cancer; SNP: single nucleotide polymorphism; OR: odds ratio; 95% CI: 95% confidence interval; BMI: mass body index

Stratified analyses were performed based on demographic features (sex, age, BMI, cigarette, and alcohol consumption) to investigate the combined effects of *MTF1* polymorphisms and these factors on LC risk, as shown in Fig. 1 and Supplementary Table 2.

*Gender*: *MTF1* rs28411034 (dominant and log-additive) and rs3748682 (codominant, dominant, and log-additive) were correlated with LC risk in males.

*Age:* No significant association of these SNPs with LC risk was observed in the subjects aged ≤ 50 years, subjects aged 61–70 years, and subjects aged > 70 years. We found that rs473279 (codominant and recessive) was associated with the higher LC risk in the subjects aged 51–60 years.

*BMI*: Rs28411034 (codominant, dominant, recessive, and log-additive) and rs3748682 (codominant, dominant, and log-additive) might be conferred to LC risk in the subjects with BMI ≥ 24 kg/m^2^.

*Smoking*: The risk-increasing association of rs28411034 (dominant and log-additive) and rs3748682 (codominant, dominant, and log-additive) with LC risk was also observed in smokers.

*Drinking*: Among drinkers, rs28411034 (codominant, dominant, and log-additive) and rs3748682 (codominant, dominant: and log-additive) might confer the increased LC risk. Whereas, rs28411352 (log-additive) was a protective factor against the susceptibility to LC in drinkers.

### Relationship of MTF1 variants to clinical characteristics of LC patients in subgroup analysis

The association between *MTF1* variants and clinical features of LC patients was examined in subgroups (Table 4 and Supplementary Table 3). Stratified by histological type (Table 4), rs28411034 (codominant, dominant, and log-additive) and rs3748682 (codominant, dominant, and log-additive) contributed to the increasing lung squamous carcinoma risk, but not lung adenocarcinoma. No noteworthy association of *MTF1* polymorphisms with stage and lymph node metastasis of LC patients was found (Supplementary Table 3).

**Table 4:** The association of *MTF1* genetic polymorphisms and pathology type risk of lung cancer

SNP ID	Model	Genotype	Control	Adenocarcinoma	OR (95% CI)	*P*-value	Squamous carcinoma	OR (95% CI)	*P*-value
rs28411034	Codominant	G/G	333 (49.7%)	154 (49.5%)	1	0.780	80 (37.7%)	1	0.003
		G/A	291 (43.4%)	132 (42.4%)	0.94 (0.70–1.24)		112 (52.8%)	**1.71 (1.22–2.39)**	
		A/A	46 (6.9%)	25 (8%)	1.12 (0.66–1.90)		20 (9.4%)	**2.00 (1.10–3.65)**	
	Dominant	G/G	333 (49.7%)	154 (49.5%)	1	0.770	80 (37.7%)	1	7.00E-04
		G/A-A/A	337 (50.3%)	157 (50.5%)	0.96 (0.73–1.26)		132 (62.3%)	**1.75 (1.26–2.42)**	
	Recessive	G/G-G/A	624 (93.1%)	286 (92%)	1	0.590	192 (90.6%)	1	0.16
		A/A	46 (6.9%)	25 (8%)	1.15 (0.69–1.92)		20 (9.4%)	1.52 (0.86–2.69)	
	Log-additive	–-	–-	–-	1.00 (0.80–1.24)	1.000	–-	**1.53 (1.19–1.97)**	0.001
rs3748682	Codominant	T/T	336 (50.4%)	154 (49.5%)	1	0.820	80 (37.7%)	1	0.001
		T/C	283 (42.4%)	131 (42.1%)	0.96 (0.72–1.27)		112 (52.8%)	**1.81 (1.29–2.54)**	
		C/C	48 (7.2%)	26 (8.4%)	1.13 (0.67–1.90)		20 (9.4%)	**1.91 (1.05–3.48)**	
	Dominant	T/T	336 (50.4%)	154 (49.5%)	1	0.890	80 (37.7%)	1	2.00E-04
		T/C–C/C	331 (49.6%)	157 (50.5%)	0.98 (0.75–1.29)		132 (62.3%)	**1.83 (1.32–2.53)**	
	Recessive	T/T-T/C	619 (92.8%)	285 (91.6%)	1	0.580	192 (90.6%)	1	0.24
		C/C	48 (7.2%)	26 (8.4%)	1.15 (0.70–1.91)		20 (9.4%)	1.41 (0.80–2.49)	
	Log-additive	–-	–-	–-	1.01 (0.82–1.26)	0.900	–-	**1.54 (1.20–1.98)**	7.00E-04

*p* values were computed by logistic regression analysis with adjustments for age, gender, smoking, drinking and BMI

Bold data indicate statistical significance (*p* < 0.05)

*SNP* single nucleotide polymorphism, *OR *odds ratio, *CI *confidence interval

### FPRP analysis

FPRP analysis (Table 5) exhibited the positive results of rs28411034 and rs3748682 for LC susceptibility in the overall analysis with 0.1 prior probability level and FPRP < 0.2. The effects of rs28411034 and rs3748682 on LC risk in men, BMI 24 kg/m^2^, drinkers, and lung cancer patients were consistent with a significant association of FPRP levels less than 0.2 at a prior probability level of 0.1. In addition, rs3748682 was also significantly associated with a positive outcome for LC risk in smokers, with an FPRP value of < 0.2 despite a prior probability level of 0.1.

**Table 5:** False-positive report probability for the associations of *MTF1* variants with the risk of lung cancer

Group/ SNPs ID	Model	OR (95% CI)	Prior probability				
			0.25	0.1	0.01	0.001	0.0001
Overall							
rs28411034	Dominant	1.26 (1.01–1.56)	**0.097**	0.244	0.780	0.973	0.997
	Log-additive	1.22 (1.03–1.45)	**0.068**	**0.179**	0.706	0.960	0.996
rs3748682	Codominant	1.28 (1.02–1.60)	**0.090**	0.228	0.765	0.970	0.997
	Dominant	1.30 (1.05–1.62)	**0.061**	**0.163**	0.682	0.956	0.995
	Log-additive	1.24 (1.04–1.47)	**0.039**	**0.108**	0.570	0.931	0.993
**Males**							
rs28411034	Codominant	1.33 (1.02–1.74)	**0.122**	0.294	0.821	0.979	0.998
	Dominant	1.36 (1.05–1.76)	**0.070**	**0.185**	0.713	0.962	0.996
	Log-additive	1.28 (1.04–1.57)	**0.054**	**0.146**	0.653	0.950	0.995
rs3748682	Codominant	1.41 (1.08–1.85)	**0.055**	**0.150**	0.659	0.951	0.995
	Dominant	1.43 (1.10–1.85)	**0.029**	**0.083**	0.500	0.910	0.990
	Log-additive	1.31 (1.06–1.60)	**0.026**	**0.075**	0.470	0.899	0.989
**Age at 51–60 years**							
rs473279	Codominant	2.74 (1.15–6.52)	0.222	0.461	0.904	0.990	0.999
	Recessive	2.79 (1.19–6.51)	**0.193**	0.418	0.888	0.988	0.999
**BMI ≥ 24 kg/m**^**2**^							
rs28411034	Codominant	1.41 (1.05–1.90)	**0.098**	0.247	0.783	0.973	0.997
		2.09 (1.20–3.63)	**0.182**	0.400	0.880	0.987	0.999
	Dominant	1.50 (1.12–1.99)	**0.029**	**0.082**	0.494	0.908	0.990
	Recessive	1.77 (1.04–3.02)	0.285	0.545	0.929	0.993	0.999
	Log-additive	1.43 (1.14–1.79)	**0.008**	**0.024**	0.212	0.731	0.964
rs3748682	Codominant	1.47 (1.09–1.98)	**0.057**	**0.155**	0.668	0.953	0.995
		1.95 (1.14–3.36)	0.219	0.457	0.903	0.989	0.999
	Dominant	1.54 (1.16–2.04)	**0.018**	**0.052**	0.377	0.859	0.984
	Log-additive	1.43 (1.14–1.79)	**0.008**	**0.024**	0.212	0.731	0.964
**Smokers**							
rs28411034	Dominant	1.35 (1.02–1.79)	**0.126**	0.303	0.827	0.980	0.998
	Log-additive	1.27 (1.01–1.60)	**0.122**	0.294	0.821	0.979	0.998
rs3748682	Codominant	1.49 (1.11–2.00)	**0.044**	**0.121**	0.603	0.939	0.994
	Dominant	1.48 (1.11–1.97)	**0.039**	**0.108**	0.571	0.931	0.993
	Log-additive	1.32 (1.05–1.66)	**0.058**	**0.155**	0.669	0.953	0.995
**Drinkers**							
rs28411034	Codominant	1.64 (1.19–2.28)	**0.032**	**0.089**	0.520	0.916	0.991
	Dominant	1.63 (1.19–2.23)	**0.022**	**0.063**	0.425	0.882	0.987
	Log-additive	1.40 (1.10–1.79)	**0.030**	**0.085**	0.504	0.911	0.990
rs28411352	Log-additive	0.73 (0.55–0.97)	**0.109**	0.269	0.802	0.976	0.998
rs3748682	Codominant	1.85 (1.33–2.56)	**0.006**	**0.018**	**0.165**	0.666	0.952
	Dominant	1.79 (1.31–2.45)	**0.006**	**0.018**	**0.169**	0.673	0.954
	Log-additive	1.47 (1.15–1.87)	**0.009**	**0.026**	0.230	0.751	0.968
**Squamous carcinoma**							
rs28411034	Codominant	1.71 (1.22–2.39)	**0.022**	**0.064**	0.430	0.884	0.987
		2.00 (1.10–3.65)	**0.100**	0.249	0.785	0.974	0.997
	Dominant	1.75 (1.26–2.42)	**0.012**	**0.035**	0.287	0.803	0.976
	Log-additive	1.53 (1.19–1.97)	**0.007**	**0.020**	**0.180**	0.689	0.957
rs3748682	Codominant	1.81 (1.29–2.54)	**0.013**	**0.037**	0.300	0.812	0.977
		1.91 (1.05–3.48)	**0.156**	0.357	0.859	0.984	0.998
	Dominant	1.83 (1.32–2.53)	**0.007**	**0.020**	**0.181**	0.690	0.957
	Log-additive	1.54 (1.20–1.98)	**0.005**	**0.016**	**0.152**	0.644	0.948

The false-positive report probability threshold level was set at 0.2, and Bold represents that noteworthy findings are presented

*SNP* single nucleotide polymorphism, *OR *odds ratio, *95% CI *95% confidence interval, *BMI *mass body index

### SNP-SNP interaction in the risk of LC

**Fig. 2:**
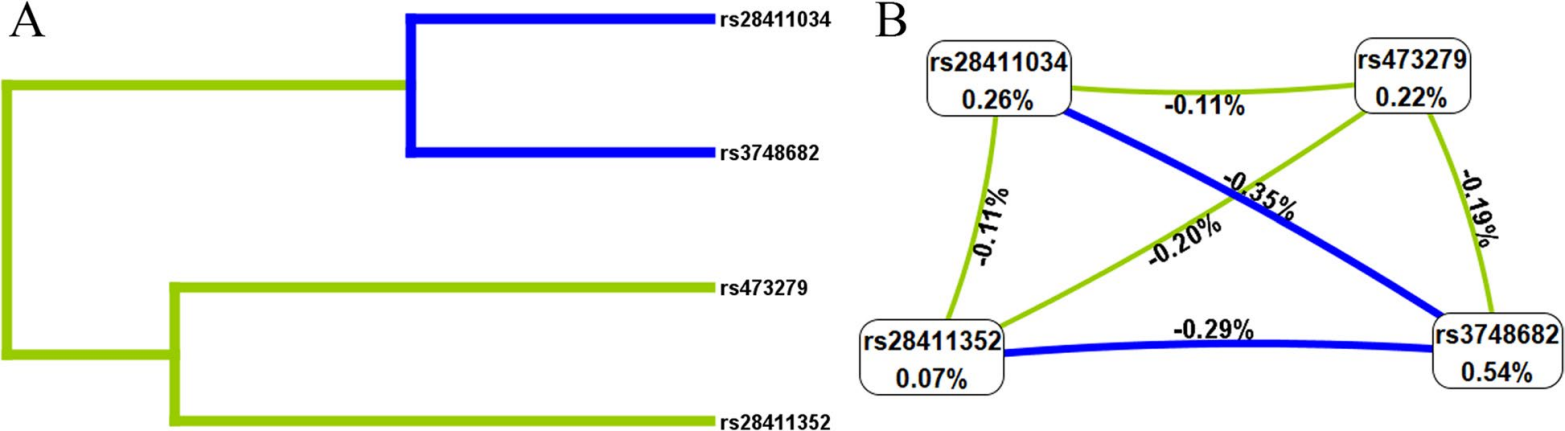
SNP-SNP interaction dendrogram (**a**) and Fruchterman-Reingold (**b**). Green and blue represent redundancy or association. Values in nodes represent the information gains of individual attribute (main effects). Values between nodes are information gains of each pair of attributes (interaction effects). SNP: single nucleotide polymorphism

The results of MDR analysis for SNP-SNP interactions showed that rs3748682 was the best single-locus model (cross-validation consistency [CVC]: 10/10; testing balanced accuracy: 0.5328). The best multi-locus model was the combined mode of rs473279, rs28411034, and rs3748682 (CVC: 10/10; testing balanced accuracy: 0.5463, Table 6). The dendrogram (Fig. 2A) showed that these loci with strong interactions were close together on the branches, while these loci with weak interactions were far apart from each other. The Fruchterman-Reingold plot (Fig. 2B) displayed that rs3748682 was the most significant single-locus factor for LC susceptibility, with an information gain of 0.54%.

**Table 6:** MDR analysis of SNP-SNP interactions

Model	Training Bal. Acc	Testing Bal. Acc	CVC
rs3748682	0.5328	0.5328	10/10
rs473279,rs3748682	0.5433	0.5425	10/10
rs473279,rs28411034,rs3748682	0.5485	0.5463	10/10
rs473279,rs28411034,rs28411352,rs3748682	0.5502	0.5172	10/10

*MDR* multifactor dimensionality reduction, *SNP *single nucleotide polymorphism, *Bal. Acc.* balanced accuracy, *CVC *cross–validation consistency

### Association between MTF1 haplotypes and the risk of LC

**Fig. 3:**
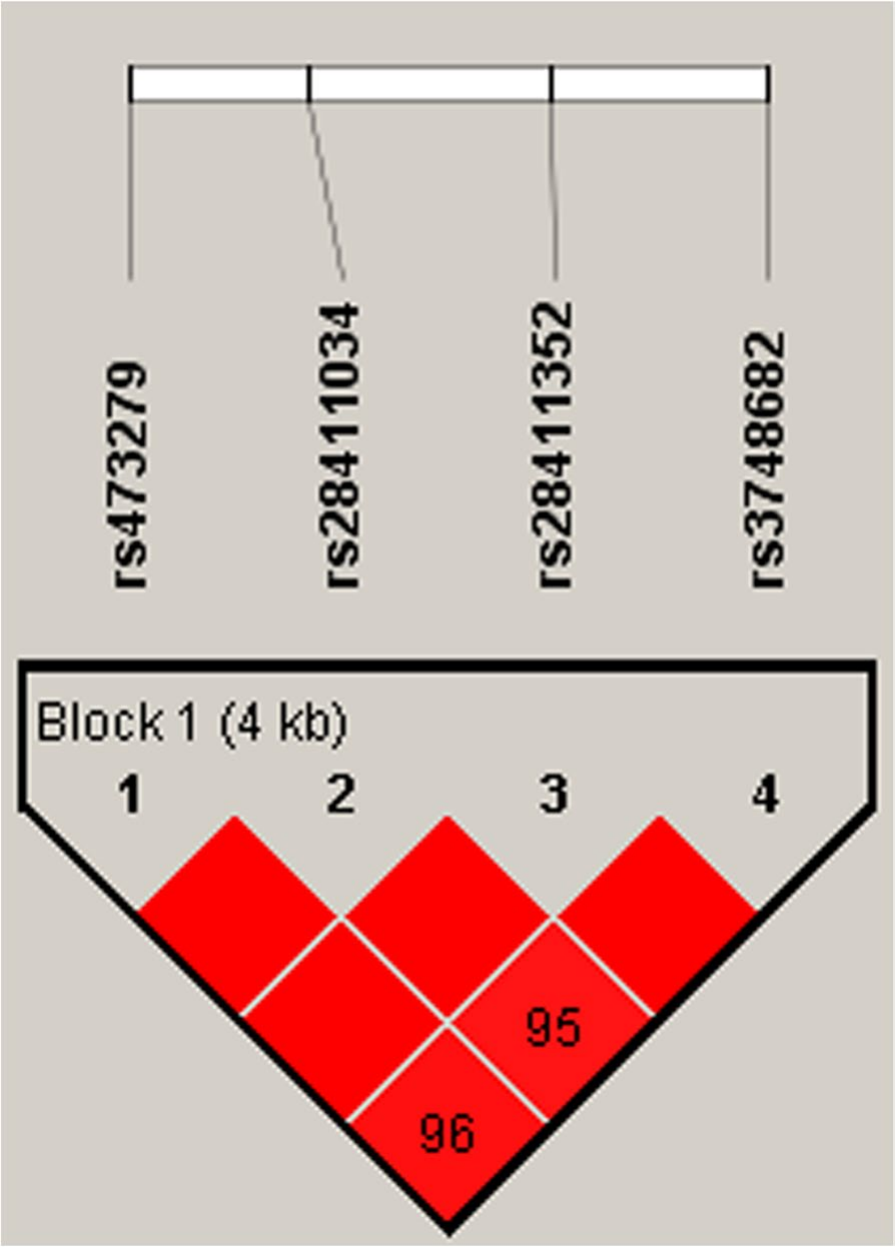
Haplotype block map for eight SNPs in the *MTF1* gene. The numbers inside the diamonds indicate the D′ for pairwise analyses

Moreover, LD and haplotype analysis was performed to estimate the association between *MTF1* haplotypes and the risk of LC. As shown in Fig. 3, a high LD block was composed of four *MTF1* polymorphisms (rs473279, rs28411034, rs28411352, rs3748682) which formed four haplotypes (C_rs473279_A_rs28411034_C_rs28411352_C_rs3748682_, T_rs473279_G_rs28411034_C_rs28411352_T_rs3748682_, C_rs473279_G_rs28411034_C_rs28411352_ T_rs3748682_, and C_rs473279_G_rs28411034_T_rs28411352_T_rs3748682_). Furthermore, the haplotype frequency distribution was shown in Table 7. We noted that there was a significant association of C_rs473279_G_rs28411034_C_rs28411352_T_rs3748682_ (OR 0.76, 95% CI 0.61–0.94, *p* = 0.013) and C_rs473279_G_rs28411034_T_rs28411352_T_rs3748682_ (OR 0.79, 95% CI 0.63–0.99, *p* = 0.045) haplotype with the reduced LC risk.

**Table 7:** Haplotype analysis for the effect of *MTF1* haplotypes on the risk of LC

Haplotype	Frequency		Crude analysis		Adjusted by age	
Block	Control	Case	OR (95% CI)	p	OR (95% CI)	p
CACC	0.273	0.284	1	–-	1	–-
TGCT	0.229	0.202	0.89 (0.72—1.10)	0.270	0.89 (0.72—1.10)	0.270
CGCT	0.203	0.188	0.76 (0.61—0.94)	**0.013**	0.76 (0.61—0.94)	**0.013**
CGTT	0.273	0.284	0.79 (0.63—0.99)	**0.045**	0.79 (0.63—0.99)	**0.045**

*MTF1* block comprises the four closely linked SNPs (rs473279, rs28411034, rs28411352, and rs3748682)

*p* values were computed by logistic regression analysis with adjustments for age, gender, smoking, drinking and BMI

Bold data indicate statistical significance (*p* < 0.05)

*OR* odds ratio, *CI* confidence interval

## Discussion

In our study, we first reported that rs28411034 and rs3748682 tended to have a higher LC susceptibility overall among the Chinese Han population. In the subgroup analysis of demographic characteristics, the effect of rs28411034 and rs3748682 on LC susceptibility was found in males, subjects with BMI ≥ 24 kg/m^2^, smokers, drinkers, and patients with lung squamous carcinoma. Besides, rs28411352 showed protective effect for reduced LC risk in drinkers. These results might contribute to understanding the pathogenesis of the *MTF1* gene in LC progression.

MTF1 is an important transcription factor for heavy metal reactions and is associated with the reduction of oxidative and hypoxic stress in cells [26]. In the presence of p53 in breast cancer cells, *MTF1* can be activated by zinc and copper [27]. Previously, *MTF1* knockout reduced the proliferation, migration, and invasion of two types of ovarian cancer cells [12]. *MTF1* is reported to be related to angiogenesis, and cell invasion [14]. Moreover, in vitro experiments have shown that *MTF1* knockdown prevents liver cancer cell proliferation, and promotes cell death [14]. The higher expression of *MTF1* may predict a better prognosis for LC patients after chemotherapy [14]. Lung adenocarcinoma cells lacking *MTF1* are more sensitive to oxidative stress [15]. Little has been reported about the contribution of *MTF1* variants to the susceptibility of tumors. This study is the first to show that rs28411034 and rs3748682 are related to the increased LC susceptibility in the Chinese Han population. Bioinformatics analysis suggested that the possible function of these SNPs might be related to SiPhy cons, promoter/ enhancer histone marks, DNAse, proteins bound, GRASP QTL hits, selected eQTL hits, transcription factor binding, and/or chromatin accessibility peak. Based on the GTEx Portal database, the genotypes of rs473279, rs28411034, rs28411352, and rs3748682 in *MTF1* were associated with the mRNA expression in lung tissue. All these SNPs were located in the 3'-UTR region of *MTF1* gene. We hypothesize that these SNPs may be related to miRNA binding sites, potentially affecting the expression levels of the *MTF1* gene by altering the binding efficiency of miRNA. Our findings imply that these polymorphisms could be implicated in the carcinogenesis of LC by potentially modulating the expression or functionality of the *MTF1* gene, thereby laying a theoretical foundation for future mechanistic investigations.

With the population aging, LC incidence is on the rise [28]. Internationally, the incidence of LC is higher in men than in women [29, 30]. Recent studies have shown that in most countries, morbidity among women is increasing, while morbidity and mortality among men are declining [31]. These studies suggest that age and sex are confounding factors in the genetic association with LC. *MTF1* rs28411034 and rs3748682 were related to LC risk in males. Moreover, rs473279 was associated with an increased LC susceptibility in subjects aged 51–60 years. BMI is a risk factor for LC and is negatively correlated with LC risk [32]. According to the Chinese BMI classification [33, 34], overweight was defined as ≥ 24 kg/m^2^. Therefore, we used BMI of 24 kg/m^2^ as a cut-off. Here, we found that rs28411034 and rs3748682 may contribute to LC risk in the subjects with BMI ≥ 24 kg/m^2^. Smoking is a well-known major risk factor for LC [35]. The role of alcohol consumption in LC occurrence is controversial. Some studies have shown that alcohol consumption increases LC risk [36], and others displayed that moderate alcohol consumption is a protective factor in LC development [37]. Stratified results showed that rs28411034 and rs3748682 were associated with LC risk in smokers and drinkers. Moreover, rs28411352 was related to the reduced LC risk in drinkers. Our study demonstrates the synergistic role of *MTF1* polymorphisms and demographic characteristics in the occurrence of LC. Our results propose that the association between *MTF1* polymorphism and LC risk are age-, sex-, BMI-, smoking-, and drinking-specific.

Lung cancer, a multifaceted condition influenced by genetic and environmental factors, may have its risk factors uncovered through polygenic or SNP-SNP interaction studies. Notably, the MDR method is recognized for its effectiveness in identifying SNP-SNP/gene–gene interactions in the absence of individual gene effects, which is crucial in case–control studies of complex diseases [38] In this study, we applied MDR to analyze the interactions among four specific SNPs. Our findings indicate that rs3748682 was the best single-locus model and the best multi-locus model was the combined mode of rs473279, rs28411034, and rs3748682.

Haplotypes, as fundamental genetic variations and units of inheritance, can influence phenotypes either directly by altering promoter activity and protein structure or indirectly through linkage with nearby untyped causal variations [39]. Consequently, haplotype association studies are vital for elucidating the etiology of complex phenotypes. In our research, haplotype analysis displayed the significant association of C_rs473279_G_rs28411034_C_rs28411352_T_rs3748682_ and C_rs473279_G_rs28411034_T_rs28411352_T_rs3748682_ haplotypes with the reduced LC risk. These hinted us that *MTF1* haplotypes could be a potential risk factor for LC occurrence.

There are some limitations to our study. First, all the samples were from one hospital, so there was some selection bias and it was not representative of the entire population. Therefore, further large-scale genetic studies in different populations are required to verify our findings. Second, our study only assesses the association between four SNPs in *MTF1* and LC risk, and a large number of loci remain to be studied. Third, this study discovered that rs28411034 and rs3748682 were related to LC risk, but their specific mechanisms in lung carcinogenesis need to be further explored in a complete functional experiment. Fourth, none of the SNPs was significant after the Bonferroni correction (*p* < 0.05/(4 × 4 × 5)). This may be in that the Bonferroni correction adjusts the value of alpha based on the number of tests performed and is thus conservative; in some cases, truly significant differences may be deemed non-significant as a result of type II errors. Therefore, we did not use the Bonferroni correction in our study.Whatever, this study has exploratory value for the relationship between *MTF1* polymorphisms and the risk of LC. Our findings need to be further confirmed in a larger sample.

## Conclusion

Taken together, our study suggests that *MTF1* rs28411034 and rs3748682 tended to be associated with increased LC susceptibility among the Chinese Han population, especially in males, subjects with BMI ≥ 24 kg/m^2^, smokers, drinkers, and patients with lung squamous carcinoma. These findings help to shed light on the underlying mechanism of the *MTF1* gene in LC progression. However, functional studies and larger patient population studies are needed to confirm our conclusions.

### Supplementary Information

Supplementary Material 1: Suppl_Figure 1. The violin plot for the association between the genotypes of *MTF1* variants and the mRNA expression in the lung tissue. Data were from GTEx Portal database (https://gtexportal.org/home/).

Supplementary Material 2.

## Data Availability

All data generated or analyzed during this study are included in this published article. The datasets generated and/or analysed during the current study are available in the Zenodo repository, https://zenodo.org/record/8384945.
