# Supplementary figures and images for: MTF1 genetic variants are associated with lung cancer risk in the Chinese Han population
Source: BMC Cancer. 2024 Jun 28;24:778. doi: 10.1186/s12885-024-12516-y (PMC11212402; doi:10.1186/s12885-024-12516-y)

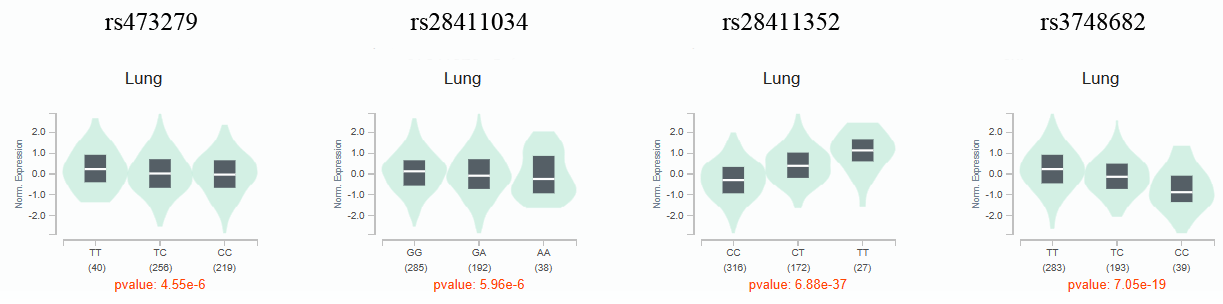

Supplement: Supplementary file 1 — Supplementary Material 1: Suppl_Figure 1. The violin plot for the association between the genotypes of MTF1 variants and the mRNA expression in the lung tissue. Data were from GTEx Portal database (https://gtexportal.org/home/). [file 12885_2024_12516_MOESM1_ESM.tif]
