# Supplementary material for: MTF1 genetic variants are associated with lung cancer risk in the Chinese Han population
Source: BMC Cancer. 2024 Jun 28;24:778. doi: 10.1186/s12885-024-12516-y (PMC11212402; doi:10.1186/s12885-024-12516-y)
Supplement: Supplementary file 2 — Supplementary Material 2. [file 12885_2024_12516_MOESM2_ESM.docx]

**Supplementary Table 1** The detail of PCR primers and UEP sequence for candidate SNPs in *MTF1*

| SNP ID | First primer(5'-3') | Second primer (5'-3') | UEP direction | UEP SEQ (5'-3') |
| --- | --- | --- | --- | --- |
| rs473279 | ACGTTGGATGACAACGAAGGATGTAGACTG | ACGTTGGATGAAGTCAGAGGCTTTCTCTTG | F | gagagGGATGTAGACTGTTGAGTCAAA |
| rs28411034 | ACGTTGGATGTGGCCTGGCAGCGTTTTTC | ACGTTGGATGCATAGATTGAGCGGTTGCTG | R | agcgCAATCAGCCCTGTGCTGTGAGA |
| rs28411352 | ACGTTGGATGAACTCATGCTCAGCAGTTGG | ACGTTGGATGGCTTCTGTTGACCATTGACC | F | ACTGGAAACCTCCTGC |
| rs3748682 | ACGTTGGATGTCTTTCTCTCGGTTGGTTGC | ACGTTGGATGCCACAAGTTCACTCTAGGAC | R | gggtTGAATTTTCCTTGGGGAAC |

SNP, Single nucleotide polymorphism; UEP, Unextended mini sequencing primer; SEQ, sequence.

**Supplementary Table 2** The association between *MTF1* polymorphisms and the susceptibility to lung cancer in the stratification of demographic characteristics

| SNP ID | Model | Genotype | Control | Case | OR (95% CI) | P-value | Control | Case | OR (95% CI) | P-value |
| --- | --- | --- | --- | --- | --- | --- | --- | --- | --- | --- |
| **Sex** |  |  | **Males** | | | | **Females** | | | |
| rs28411034 | Codominant | G/G | 241 (51.9%) | 211 (44.3%) | 1 | 0.055 | 92 (44.7%) | 85 (43.8%) | 1 | 0.770 |
|  |  | G/A | 192 (41.4%) | 223 (46.9%) | **1.33 (1.02-1.74)** |  | 99 (48.1%) | 91 (46.9%) | 0.99 (0.65-1.49) |  |
|  |  | A/A | 31 (6.7%) | 42 (8.8%) | 1.53 (0.93-2.53) |  | 15 (7.3%) | 18 (9.3%) | 1.30 (0.61-2.75) |  |
|  | Dominant | G/G | 241 (51.9%) | 211 (44.3%) | 1 | 0.019 | 92 (44.7%) | 85 (43.8%) | 1 | 0.890 |
|  |  | G/A-A/A | 223 (48.1%) | 265 (55.7%) | **1.36 (1.05-1.76)** |  | 114 (55.3%) | 109 (56.2%) | 1.03 (0.69-1.53) |  |
|  | Recessive | G/G-G/A | 433 (93.3%) | 434 (91.2%) | 1 | 0.240 | 191 (92.7%) | 176 (90.7%) | 1 | 0.470 |
|  |  | A/A | 31 (6.7%) | 42 (8.8%) | 1.33 (0.82-2.17) |  | 15 (7.3%) | 18 (9.3%) | 1.31 (0.64-2.69) |  |
|  | Log-additive | --- | --- | --- | **1.28 (1.04-1.57)** | 0.018 | --- | --- | 1.07 (0.78-1.47) | 0.670 |
| rs3748682 | Codominant | T/T | 245 (53%) | 211 (44.3%) | 1 | 0.025 | 91 (44.4%) | 84 (43.3%) | 1 | 0.780 |
|  |  | T/C | 184 (39.8%) | 222 (46.6%) | **1.41 (1.08-1.85)** |  | 99 (48.3%) | 92 (47.4%) | 1.00 (0.66-1.51) |  |
|  |  | C/C | 33 (7.1%) | 43 (9%) | 1.50 (0.92-2.46) |  | 15 (7.3%) | 18 (9.3%) | 1.30 (0.61-2.75) |  |
|  | Dominant | T/T | 245 (53%) | 211 (44.3%) | 1 | 0.007 | 91 (44.4%) | 84 (43.3%) | 1 | 0.860 |
|  |  | T/C-C/C | 217 (47%) | 265 (55.7%) | **1.43 (1.10-1.85)** |  | 114 (55.6%) | 110 (56.7%) | 1.04 (0.69-1.55) |  |
|  | Recessive | T/T-T/C | 429 (92.9%) | 433 (91%) | 1 | 0.310 | 190 (92.7%) | 176 (90.7%) | 1 | 0.480 |
|  |  | C/C | 33 (7.1%) | 43 (9%) | 1.28 (0.80-2.06) |  | 15 (7.3%) | 18 (9.3%) | 1.30 (0.63-2.67) |  |
|  | Log-additive | --- | --- | --- | **1.31 (1.06-1.60)** | 0.010 | --- | --- | 1.08 (0.78-1.48) | 0.650 |
| **Age** |  |  | **≤ 50 years** | | | | **51-60 years** | | | |
| rs473279 | Codominant | C/C | 56 (53.3%) | 67 (55.4%) | 1 | 0.470 | 109 (52.7%) | 104 (51%) | 1 | **0.044** |
|  |  | C/T | 37 (35.2%) | 46 (38%) | 1.05 (0.60-1.86) |  | 90 (43.5%) | 80 (39.2%) | 0.96 (0.64-1.44) |  |
|  |  | T/T | 12 (11.4%) | 8 (6.6%) | 0.57 (0.21-1.52) |  | 8 (3.9%) | 20 (9.8%) | **2.74 (1.15-6.52)** |  |
|  | Dominant | C/C | 56 (53.3%) | 67 (55.4%) | 1 | 0.810 | 109 (52.7%) | 104 (51%) | 1 | 0.620 |
|  |  | C/T-T/T | 49 (46.7%) | 54 (44.6%) | 0.94 (0.55-1.60) |  | 98 (47.3%) | 100 (49%) | 1.11 (0.75-1.63) |  |
|  | Recessive | C/C-C/T | 93 (88.6%) | 113 (93.4%) | 1 | 0.230 | 199 (96.1%) | 184 (90.2%) | 1 | **0.013** |
|  |  | T/T | 12 (11.4%) | 8 (6.6%) | 0.56 (0.22-1.45) |  | 8 (3.9%) | 20 (9.8%) | **2.79 (1.19-6.51)** |  |
|  | Log-additive | --- | --- | --- | 0.86 (0.57-1.30) | 0.480 | --- | --- | 1.25 (0.91-1.72) | 0.160 |
|  |  |  | **61-70 years** | | | | **> 70 years** | | | |
| rs473279 | Codominant | C/C | 126 (46.7%) | 125 (50%) | 1 | 0.670 | 48 (55.8%) | 45 (47.4%) | 1 | 0.390 |
|  |  | C/T | 125 (46.3%) | 106 (42.4%) | 0.86 (0.60-1.23) |  | 35 (40.7%) | 45 (47.4%) | 1.43 (0.76-2.67) |  |
|  |  | T/T | 19 (7%) | 19 (7.6%) | 1.03 (0.52-2.06) |  | 3 (3.5%) | 5 (5.3%) | 2.18 (0.47-10.16) |  |
|  | Dominant | C/C | 126 (46.7%) | 125 (50%) | 1 | 0.470 | 48 (55.8%) | 45 (47.4%) | 1 | 0.210 |
|  |  | C/T-T/T | 144 (53.3%) | 125 (50%) | 0.88 (0.62-1.24) |  | 38 (44.2%) | 50 (52.6%) | 1.48 (0.80-2.73) |  |
|  | Recessive | C/C-C/T | 251 (93%) | 231 (92.4%) | 1 | 0.750 | 83 (96.5%) | 90 (94.7%) | 1 | 0.420 |
|  |  | T/T | 19 (7%) | 19 (7.6%) | 1.11 (0.57-2.17) |  | 35 (40.7%) | 45 (47.4%) | 1.34 (0.72-2.46) |  |
|  | Log-additive | --- | --- | --- | 0.94 (0.71-1.24) | 0.650 | --- | --- | 1.44 (0.85-2.45) | 0.170 |
| **BMI** |  | | **≥ 24 kg/m^2^** | | | | **< kg/m^2^** | | | |
| rs28411034 | Codominant | G/G | 206 (52.4%) | 167 (43.1%) | 1 | 0.008 | 127 (45.9%) | 129 (45.6%) | 1 | 0.890 |
|  |  | G/A | 163 (41.5%) | 181 (46.8%) | **1.41 (1.05-1.90)** |  | 128 (46.2%) | 133 (47%) | 1.08 (0.76-1.53) |  |
|  |  | A/A | 24 (6.1%) | 39 (10.1%) | **2.09 (1.20-3.63)** |  | 22 (7.9%) | 21 (7.4%) | 0.98 (0.51-1.88) |  |
|  | Dominant | G/G | 206 (52.4%) | 167 (43.1%) | 1 | 0.006 | 127 (45.9%) | 129 (45.6%) | 1 | 0.720 |
|  |  | G/A-A/A | 187 (47.6%) | 220 (56.9%) | **1.50 (1.12-1.99)** |  | 150 (54.1%) | 154 (54.4%) | 1.06 (0.76-1.49) |  |
|  | Recessive | G/G-G/A | 369 (93.9%) | 348 (89.9%) | 1 | 0.033 | 255 (92.1%) | 262 (92.6%) | 1 | 0.840 |
|  |  | A/A | 24 (6.1%) | 39 (10.1%) | **1.77 (1.04-3.02)** |  | 22 (7.9%) | 21 (7.4%) | 0.94 (0.50-1.76) |  |
|  | Log-additive | --- | --- | --- | **1.43 (1.14-1.79)** | 0.002 | --- | --- | 1.03 (0.79-1.35) | 0.840 |
| rs3748682 | Codominant | T/T | 207 (52.8%) | 166 (42.9%) | 1 | 0.007 | 129 (46.9%) | 129 (45.6%) | 1 | 0.810 |
|  |  | T/C | 159 (40.6%) | 182 (47%) | **1.47 (1.09-1.98)** |  | 124 (45.1%) | 132 (46.6%) | 1.12 (0.79-1.59) |  |
|  |  | C/C | 26 (6.6%) | 39 (10.1%) | **1.95 (1.14-3.36)** |  | 22 (8%) | 22 (7.8%) | 1.03 (0.54-1.98) |  |
|  | Dominant | T/T | 207 (52.8%) | 166 (42.9%) | 1 | 0.003 | 129 (46.9%) | 129 (45.6%) | 1 | 0.550 |
|  |  | T/C-C/C | 185 (47.2%) | 221 (57.1%) | **1.54 (1.16-2.04)** |  | 146 (53.1%) | 154 (54.4%) | 1.11 (0.79-1.56) |  |
|  | Recessive | T/T-T/C | 366 (93.4%) | 348 (89.9%) | 1 | 0.066 | 253 (92%) | 261 (92.2%) | 1 | 0.940 |
|  |  | C/C | 26 (6.6%) | 39 (10.1%) | 1.62 (0.96-2.73) |  | 22 (8%) | 22 (7.8%) | 0.98 (0.52-1.82) |  |
|  | Log-additive | --- | --- | --- | **1.43 (1.14-1.79)** | 0.002 | --- | --- | 1.06 (0.81-1.39) | 0.660 |
| **Smoking** |  |  | **Yes** | | | | **No** | | | |
| rs28411034 | Codominant | G/G | 201 (52.1%) | 180 (44.4%) | 1 | 0.110 | 132 (46.5%) | 116 (43.8%) | 1 | 0.640 |
|  |  | G/A | 161 (41.7%) | 191 (47.2%) | 1.33 (0.99-1.79) |  | 130 (45.8%) | 123 (46.4%) | 1.02 (0.71-1.46) |  |
|  |  | A/A | 24 (6.2%) | 34 (8.4%) | 1.47 (0.84-2.60) |  | 22 (7.8%) | 26 (9.8%) | 1.35 (0.71-2.55) |  |
|  | Dominant | G/G | 201 (52.1%) | 180 (44.4%) | 1 | 0.038 | 132 (46.5%) | 116 (43.8%) | 1 | 0.720 |
|  |  | G/A-A/A | 185 (47.9%) | 225 (55.6%) | **1.35 (1.02-1.79)** |  | 152 (53.5%) | 149 (56.2%) | 1.07 (0.75-1.51) |  |
|  | Recessive | G/G-G/A | 362 (93.8%) | 371 (91.6%) | 1 | 0.370 | 262 (92.2%) | 239 (90.2%) | 1 | 0.350 |
|  |  | A/A | 24 (6.2%) | 34 (8.4%) | 1.29 (0.74-2.23) |  | 22 (7.8%) | 26 (9.8%) | 1.34 (0.73-2.46) |  |
|  | Log-additive | --- | --- | --- | **1.27 (1.01-1.60)** | 0.041 | --- | --- | 1.10 (0.84-1.44) | 0.490 |
| rs3748682 | Codominant | T/T | 207 (53.9%) | 179 (44.2%) | 1 | 0.026 | 129 (45.6%) | 116 (43.8%) | 1 | 0.570 |
|  |  | T/C | 151 (39.3%) | 192 (47.4%) | **1.49 (1.11-2.00)** |  | 132 (46.6%) | 122 (46%) | 0.97 (0.67-1.39) |  |
|  |  | C/C | 26 (6.8%) | 34 (8.4%) | 1.42 (0.81-2.48) |  | 22 (7.8%) | 27 (10.2%) | 1.35 (0.72-2.55) |  |
|  | Dominant | T/T | 207 (53.9%) | 179 (44.2%) | 1 | 0.007 | 129 (45.6%) | 116 (43.8%) | 1 | 0.910 |
|  |  | T/C-C/C | 177 (46.1%) | 226 (55.8%) | **1.48 (1.11-1.97)** |  | 154 (54.4%) | 149 (56.2%) | 1.02 (0.72-1.44) |  |
|  | Recessive | T/T-T/C | 358 (93.2%) | 371 (91.6%) | 1 | 0.550 | 261 (92.2%) | 238 (89.8%) | 1 | 0.300 |
|  |  | C/C | 26 (6.8%) | 34 (8.4%) | 1.18 (0.69-2.02) |  | 22 (7.8%) | 27 (10.2%) | 1.38 (0.75-2.52) |  |
|  | Log-additive | --- | --- | --- | **1.32 (1.05-1.66)** | 0.016 | --- | --- | 1.08 (0.82-1.41) | 0.580 |
| **Drinking** |  |  | **Yes** | | | | **No** | | | |
| rs28411034 | Codominant | G/G | 179 (53.9%) | 149 (42.1%) | 1 | 0.008 | 154 (45.6%) | 147 (46.5%) | 1 | 0.360 |
|  |  | G/A | 126 (38%) | 172 (48.6%) | **1.64 (1.19-2.28)** |  | 165 (48.8%) | 142 (44.9%) | 0.87 (0.63-1.21) |  |
|  |  | A/A | 27 (8.1%) | 33 (9.3%) | 1.57 (0.88-2.78) |  | 19 (5.6%) | 27 (8.5%) | 1.35 (0.71-2.56) |  |
|  | Dominant | G/G | 179 (53.9%) | 149 (42.1%) | 1 | 0.002 | 154 (45.6%) | 147 (46.5%) | 1 | 0.620 |
|  |  | G/A-A/A | 153 (46.1%) | 205 (57.9%) | **1.63 (1.19-2.23)** |  | 184 (54.4%) | 169 (53.5%) | 0.92 (0.67-1.26) |  |
|  | Recessive | G/G-G/A | 305 (91.9%) | 321 (90.7%) | 1 | 0.450 | 319 (94.4%) | 289 (91.5%) | 1 | 0.240 |
|  |  | A/A | 27 (8.1%) | 33 (9.3%) | 1.24 (0.71-2.15) |  | 19 (5.6%) | 27 (8.5%) | 1.44 (0.78-2.68) |  |
|  | Log-additive | --- | --- | --- | **1.40 (1.10-1.79)** | 0.006 | --- | --- | 1.01 (0.78-1.30) | 0.940 |
| rs28411352 | Codominant | C/C | 207 (62.4%) | 242 (68.4%) | 1 | 0.084 | 219 (64.8%) | 197 (62.3%) | 1 | 0.720 |
|  |  | C/T | 110 (33.1%) | 102 (28.8%) | 0.76 (0.54-1.07) |  | 105 (31.1%) | 107 (33.9%) | 1.15 (0.82-1.61) |  |
|  |  | T/T | 15 (4.5%) | 10 (2.8%) | 0.48 (0.21-1.11) |  | 14 (4.1%) | 12 (3.8%) | 1.03 (0.46-2.32) |  |
|  | Dominant | C/C | 207 (62.4%) | 242 (68.4%) | 1 | 0.051 | 219 (64.8%) | 197 (62.3%) | 1 | 0.440 |
|  |  | C/T-T/T | 125 (37.6%) | 112 (31.6%) | 0.72 (0.52-1.00) |  | 119 (35.2%) | 119 (37.7%) | 1.14 (0.82-1.57) |  |
|  | Recessive | C/C-C/T | 317 (95.5%) | 344 (97.2%) | 1 | 0.120 | 324 (95.9%) | 304 (96.2%) | 1 | 0.970 |
|  |  | T/T | 15 (4.5%) | 10 (2.8%) | 0.52 (0.23-1.20) |  | 14 (4.1%) | 12 (3.8%) | 0.98 (0.44-2.20) |  |
|  | Log-additive | --- | --- | --- | **0.73 (0.55-0.97)** | 0.028 | --- | --- | 1.09 (0.83-1.44) | 0.520 |
| rs3748682 | Codominant | T/T | 184 (55.6%) | 148 (41.8%) | 1 | 0.001 | 152 (45.2%) | 147 (46.5%) | 1 | 0.340 |
|  |  | T/C | 119 (36%) | 173 (48.9%) | **1.85 (1.33-2.56)** |  | 164 (48.8%) | 141 (44.6%) | 0.85 (0.62-1.18) |  |
|  |  | C/C | 28 (8.5%) | 33 (9.3%) | 1.56 (0.88-2.75) |  | 20 (6%) | 28 (8.9%) | 1.30 (0.69-2.44) |  |
|  | Dominant | T/T | 184 (55.6%) | 148 (41.8%) | 1 | 0.001 | 152 (45.2%) | 147 (46.5%) | 1 | 0.530 |
|  |  | T/C-C/C | 147 (44.4%) | 206 (58.2%) | **1.79 (1.31-2.45)** |  | 184 (54.8%) | 169 (53.5%) | 0.90 (0.66-1.24) |  |
|  | Recessive | T/T-T/C | 303 (91.5%) | 321 (90.7%) | 1 | 0.570 | 316 (94%) | 288 (91.1%) | 1 | 0.270 |
|  |  | C/C | 28 (8.5%) | 33 (9.3%) | 1.17 (0.68-2.02) |  | 20 (6%) | 28 (8.9%) | 1.41 (0.77-2.58) |  |
|  | Log-additive | --- | --- | --- | **1.47 (1.15-1.87)** | 0.002 | --- | --- | 0.99 (0.77-1.28) | 0.960 |

OR, odds ratio; CI, confidence interval.

*p* values were computed by logistic regression analysis with adjustments for age, gender, smoking, drinking or BMI.

Bold data indicate statistical significance (*p* < 0.05).

**Supplementary Table 3** The association between *MTF1* polymorphisms and the susceptibility to lung cancer by stage and lymph node metastasis

| SNP ID | Model | Genotype | Stage | | | | Lymph node metastasis | | | |
| --- | --- | --- | --- | --- | --- | --- | --- | --- | --- | --- |
|  |  |  | I-II | III-IV | OR (95% CI) | P-value | Yes | No | OR (95% CI) | P-value |
| rs473279 | Codominant | C/C | 139 (47.8%) | 202 (53.3%) | 1 | 0.230 | 178 (51.9%) | 157 (50.2%) | 1 | 0.690 |
|  |  | C/T | 126 (43.3%) | 151 (39.8%) | 0.82 (0.60-1.14) |  | 137 (39.9%) | 134 (42.8%) | 1.11 (0.80-1.54) |  |
|  |  | T/T | 26 (8.9%) | 26 (6.9%) | 0.63 (0.35-1.14) |  | 28 (8.2%) | 22 (7%) | 0.88 (0.48-1.61) |  |
|  | Dominant | C/C | 139 (47.8%) | 202 (53.3%) | 1 | 0.140 | 178 (51.9%) | 157 (50.2%) | 1 | 0.660 |
|  |  | C/T-T/T | 152 (52.2%) | 177 (46.7%) | 0.79 (0.58-1.08) |  | 165 (48.1%) | 156 (49.8%) | 1.07 (0.79-1.46) |  |
|  | Recessive | C/C-C/T | 265 (91.1%) | 353 (93.1%) | 1 | 0.200 | 315 (91.8%) | 291 (93%) | 1 | 0.560 |
|  |  | T/T | 26 (8.9%) | 26 (6.9%) | 0.69 (0.39-1.22) |  | 28 (8.2%) | 22 (7%) | 0.84 (0.47-1.51) |  |
|  | Log-additive | --- | --- | --- | 0.81 (0.63-1.03) | 0.087 | --- | --- | 1.01 (0.79-1.29) | 0.910 |
| rs28411034 | Codominant | G/G | 135 (46.4%) | 161 (42.5%) | 1 | 0.260 | 158 (46.1%) | 130 (41.5%) | 1 | 0.280 |
|  |  | G/A | 135 (46.4%) | 179 (47.2%) | 1.14 (0.82-1.57) |  | 159 (46.4%) | 150 (47.9%) | 1.16 (0.84-1.60) |  |
|  |  | A/A | 21 (7.2%) | 39 (10.3%) | 1.61 (0.90-2.90) |  | 26 (7.6%) | 33 (10.5%) | 1.56 (0.88-2.75) |  |
|  | Dominant | G/G | 135 (46.4%) | 161 (42.5%) | 1 | 0.250 | 158 (46.1%) | 130 (41.5%) | 1 | 0.220 |
|  |  | G/A-A/A | 156 (53.6%) | 218 (57.5%) | 1.20 (0.88-1.64) |  | 185 (53.9%) | 183 (58.5%) | 1.21 (0.89-1.66) |  |
|  | Recessive | G/G-G/A | 270 (92.8%) | 340 (89.7%) | 1 | 0.150 | 317 (92.4%) | 280 (89.5%) | 1 | 0.190 |
|  |  | A/A | 21 (7.2%) | 39 (10.3%) | 1.51 (0.86-2.64) |  | 26 (7.6%) | 33 (10.5%) | 1.44 (0.84-2.48) |  |
|  | Log-additive | --- | --- | --- | 1.21 (0.95-1.55) | 0.120 | --- | --- | 1.21 (0.95-1.54) | 0.120 |
| rs28411352 | Codominant | C/C | 193 (66.3%) | 246 (64.9%) | 1 | 0.730 | 214 (62.4%) | 215 (68.7%) | 1 | 0.098 |
|  |  | C/T | 87 (29.9%) | 122 (32.2%) | 1.05 (0.75-1.47) |  | 114 (33.2%) | 92 (29.4%) | 0.81 (0.58-1.13) |  |
|  |  | T/T | 11 (3.8%) | 11 (2.9%) | 0.73 (0.30-1.76) |  | 15 (4.4%) | 6 (1.9%) | 0.41 (0.15-1.07) |  |
|  | Dominant | C/C | 193 (66.3%) | 246 (64.9%) | 1 | 0.930 | 214 (62.4%) | 215 (68.7%) | 1 | 0.100 |
|  |  | C/T-T/T | 98 (33.7%) | 133 (35.1%) | 1.01 (0.73-1.41) |  | 129 (37.6%) | 98 (31.3%) | 0.76 (0.55-1.06) |  |
|  | Recessive | C/C-C/T | 280 (96.2%) | 368 (97.1%) | 1 | 0.460 | 328 (95.6%) | 307 (98.1%) | 1 | 0.078 |
|  |  | T/T | 11 (3.8%) | 11 (2.9%) | 0.72 (0.30-1.72) |  | 15 (4.4%) | 6 (1.9%) | 0.43 (0.17-1.14) |  |
|  | Log-additive | --- | --- | --- | 0.98 (0.73-1.30) | 0.870 | --- | --- | 0.75 (0.56-1.00) | 0.048 |
| rs3748682 | Codominant | T/T | 136 (46.7%) | 159 (42%) | 1 | 0.260 | 159 (46.4%) | 128 (40.9%) | 1 | 0.240 |
|  |  | T/C | 133 (45.7%) | 181 (47.8%) | 1.20 (0.86-1.66) |  | 157 (45.8%) | 152 (48.6%) | 1.22 (0.88-1.69) |  |
|  |  | C/C | 22 (7.6%) | 39 (10.3%) | 1.55 (0.87-2.78) |  | 27 (7.9%) | 33 (10.5%) | 1.53 (0.87-2.69) |  |
|  | Dominant | T/T | 136 (46.7%) | 159 (42%) | 1 | 0.170 | 159 (46.4%) | 128 (40.9%) | 1 | 0.140 |
|  |  | T/C-C/C | 155 (53.3%) | 220 (58%) | 1.25 (0.91-1.70) |  | 184 (53.6%) | 185 (59.1%) | 1.27 (0.93-1.73) |  |
|  | Recessive | T/T-T/C | 269 (92.4%) | 340 (89.7%) | 1 | 0.210 | 316 (92.1%) | 280 (89.5%) | 1 | 0.240 |
|  |  | C/C | 22 (7.6%) | 39 (10.3%) | 1.42 (0.81-2.47) |  | 27 (7.9%) | 33 (10.5%) | 1.38 (0.80-2.36) |  |
|  | Log-additive | --- | --- | --- | 1.22 (0.96-1.57) | 0.100 | --- | --- | 1.23 (0.97-1.57) | 0.093 |

SNP: single nucleotide polymorphism; OR: odds ratio; 95% CI: 95% confidence interval.

*p* values were computed by logistic regression analysis with adjustments for age, gender, smoking, drinking and BMI.
